# Estradiol-dependent and independent effects of FGF21 in obese female mice

**DOI:** 10.18699/VJGB-22-20

**Published:** 2022-03

**Authors:** T.V. Jakovleva, A.Yu. Kazantseva, A.D. Dubinina, N.Yu. Balybina, K.O. Baranov, E.N. Makarova, N.M. Bazhan

**Affiliations:** Institute of Cytology and Genetics of the Siberian Branch of the Russian Academy of Sciences, Novosibirsk, Russia; Institute of Cytology and Genetics of the Siberian Branch of the Russian Academy of Sciences, Novosibirsk, Russia; Institute of Cytology and Genetics of the Siberian Branch of the Russian Academy of Sciences, Novosibirsk, Russia; Institute of Cytology and Genetics of the Siberian Branch of the Russian Academy of Sciences, Novosibirsk, Russia; Institute of Molecular and Cellular Biology of the Siberian Branch of the Russian Academy of Sciences, Novosibirsk, Russia; Institute of Cytology and Genetics of the Siberian Branch of the Russian Academy of Sciences, Novosibirsk, Russia; Institute of Cytology and Genetics of the Siberian Branch of the Russian Academy of Sciences, Novosibirsk, Russia Novosibirsk State University, Novosibirsk, Russia

**Keywords:** FGF21, estradiol, liver, adipose tissue, food preference, gene expression, sex differences, FGF21, эстрадиол, печень, жировая ткань, пищевое предпочтение, экспрессия генов, половые различия

## Abstract

The f ibroblast growth factor 21 (FGF21) synthesized in the liver, acting as a hormone, increases insulin sensitivity and energy expenditure. FGF21 administration has potent benef icial effects on obesity and diabetes in humans, cynomolgus monkey, and rodents. The therapeutic effects of FGF21 have been studied mainly in males. They are not always manifested in females, and they are accompanied by sex-specif ic activation of gene expression in tissues. We have suggested that one of the causes of sexual dimorphism in response to FGF21 is the effect of estradiol (E2). Currently, it is not known how estradiol modif ies the pharmacological effects of FGF21. The objec tive of this study was to study the inf luence of FGF21 on metabolic characteristics, food intake, and the expression of carbohydrate and fat metabolism genes in the liver, adipose tissue, and hypothalamus in female mice with alimentary obesity and low (ovariectomy) or high (ovariectomy + E2) blood estradiol level. In ovariectomized (OVX) females, the development of obesity was induced by the consumption of a high sweet-fat diet (standard chow, lard, and cookies) for 8 weeks. We investigated the effects of FGF21 on body weight, blood levels, food preferences and gene expression in tissues when FGF21 was administered separately or in combination with E2 for 13 days. In OVX obese females, FGF21, regardless of E2-treatment, did not affect body weight, and adipose tissue weight, or glucose tolerance but increased the consumption of standard chow, reduced blood glucose levels, and suppressed its own expression in the liver (Fgf21), as well as the expression of the G6pc and Acacα genes. This study is the f irst to show the modif ication of FGF21 effects by estradiol: inhibition of FGF21-inf luence on the expression of Irs2 and Pklr in the liver and potentiation of the FGF21-stimulated expression of Lepr and Klb in the hypothalamus. In addition, when administered together with estradiol, FGF21 exerted an inhibitory effect on the expression of Cpt1α in subcutaneous white adipose tissue (scWAT), whereas no stimulating FGF21 effects on the expression of Insr and Acacβ in scWAT or inhibitory FGF21 effect on the plasma insulin level were observed. The results suggest that the absence of FGF21 effects on body and adipose tissue weights in OVX obese females and its benef icial effect on food intake and blood glucose levels are not associated with the action of estradiol. However, estradiol affects the transcriptional effects of FGF21 in the liver, white adipose tissue, and hypothalamus, which may underlie sex differences in the FGF21 effect on the expression of metabolic genes and, possibly, in pharmacological FGF21 effects

## Abstract (ru)

Синтезируемый в печени фактор роста фибробластов 21 (FGF21), действуя как гормон, повышает чувствительность к инсулину и расход энергии. Введение FGF21 оказывает мощное благотворное воздействие у людей, обезьян и грызунов с ожирением и диабетом второго типа. Терапевтические эффекты FGF21 исследованы главным образом на самцах, они не всегда проявляются у самок и сопровождаются поло-специфической активацией экспрессии генов в тканях. Мы предположили, что одной из причин полового диморфизма в ответе на FGF21 является действие эстрадиола (Е2). В настоящее время неизвестно, как эстрадиол влияет на проявление фармакологических эффектов FGF21. Задачей данного исследования было изучение влияния FGF21 на метаболические характеристики, потребление пищи и экспрессию генов углеводного и жирового обмена в печени, жировой ткани и гипоталамусе у самок мышей с алиментарным ожирением на фоне низкого (овариэктомия) и высокого (овариэктомия + Е2) уровня эстрадиола в крови. У овариэктомированных самок развитие ожирения индуцировали потреблением сладко-жирной диеты (стандартный корм, сало, печенье) в течение 8 недель. Мы оценили эффекты FGF21 на массу тела, показатели крови, выбор компонентов диеты, экспрессию генов в тканях при раздельном и совместном введении с Е2 в течение 13 дней. У овариэктомированных самок с ожирением FGF21, независимо от введения Е2, не влиял на массу тела и жировой ткани, толерантность к глюкозе, повышал потребление стандартного корма, снижал уровень глюкозы в крови, подавлял в печени собственную экспрессию (Fgf21), а также экспрессию генов G6pc и Acacα. Впервые показано влияние эстрадиола на эффекты FGF21: ингибирование FGF21-влияния на экспрессию Irs2 и Pklr в печени и потенцирование FGF21-стимулированной экспрессии Lepr и Klb в гипоталамусе. Кроме того, на фоне введения эстрадиола не проявлялось ингибирующее влияние FGF21 на уровень инсулина в крови, а в подкожной белой жировой ткани проявлялось ингибирующее влияние FGF21 на экспрессию Cpt1α и не проявлялось активирующее влияние FGF21 на экспрессию генов Insr и Acacβ. Полученные данные позволяют заключить, что у овариэктомированных самок с ожирением отсутствие эффектов FGF21 на массу тела и жировой ткани и его благотворное влияние на потребление пищи и уровень глюкозы в крови не связаны с действием эстрадиола. Однако эстрадиол влияет на транскрипционные эффекты FGF21 в печени, белом жире и гипоталамусе, что может лежать в основе половых различий в его действии на экспрессию метаболических генов и, возможно, половых различий его фармакологических эффектов.

## Introduction

Fibroblast growth factor 21 (FGF21) is synthesized in the liver, secreted into blood and acts as a hormone (Kharitonenkov et al., 2005). Its level increases significantly at metabolic stress; specifically, in the cold, fasting, and obesity (Fisher et al., 2011). FGF21 is involved in the regulation of carbohydratelipid metabolism. Its pharmacological doses improve metabolic parameters in animals and people with obesity: they increase energy expenditure and insulin sensitivity and reduce blood glucose levels (Kharitonenkov et al., 2005; Coskun et al., 2008). In addition, FGF21 affects taste preferences: it reduces the consumption of sweets and alcohol and increases protein consumption (Talukdar et al., 2016; Allard et al., 2019; Larson et al., 2019).

Currently, FGF21 and its synthetic analogues are used in designing drugs for the treatment of metabolic syndrome in obesity. However, the vast majority of preclinical studies of its pharmacological action were performed on males. Our studies of the effect of FGF21 in mice showed that its pharmacological effects in females and males might differ. In female C57Bl mice with obesity induced by the consumption of high sweet-fat diet, FGF21 reduced body weight, but, unlike males, did not affect glucose tolerance or the expression of metabolic genes in the liver or in brown adipose tissue (Bazhan et al., 2019). In obese C57Bl mice fed a mixture of high-fat and standard diets, administration of FGF21 improved some metabolic indices in mice of both sexes but induced female-specific activation of gene expression in abdominal adipose tissue (Makarova et al., 2021). In female Ay mice with genetically induced obesity, unlike males, FGF21 did not affect body weight, blood insulin levels, or POMC expression in the hypothalamus, but increased food intake and liver weight and modified the expression of metabolic genes in the liver and in white adipose tissue (Makarova et al., 2020).

The causes of the sex differences in the pharmacological effects of FGF21 are still unknown. We assumed that sex differences in responses to FGF21 were associated with the influence of estrogens. Estradiol and FGF21 esert similar effects on metabolic parameters. Estradiol, like FGF21, reduces food intake, body weight, blood glucose and insulin levels and increases glucose tolerance in ovariectomized and intact obese females (Gao et al., 2006; Thammacharoen et al., 2009). According to the available data, estradiol and FGF21 have different receptors and the same signaling pathways (Fisher et al., 2011; Vrtačnik et al., 2014). In addition, estradiol can affect the level of FGF21 in blood. The expression of Fg f 21 in the liver determines the blood hormone level and depends on the stage of the estrous cycle, positively correlating with the blood level of estradiol (Hua et al., 2018). Exogenous estradiol can also stimulate the expression of Fg f 21 in the liver and increase the blood FGF21 level (Allard et al., 2019).

We suggested that FGF21 and estradiol interact in the regulation of carbohydrate-lipid metabolism, and the pharmacological effects of FGF21 depend on the blood estradiol level. Therefore, the aim of this study was to compare the effects of FGF21 on metabolic parameters, food choice, and the expression of genes involved in carbohydrate and fat metabolism in the liver, adipose tissue, and hypothalamus in female mice with alimentary obesity and different levels of estrogenic activity.

## Materials and methods

Animals. All experiments were performed according to the Guide for the Care and Use of Laboratory Animals (1996) and the Russian National Guidelines for the Care and Use of Laboratory Animals.

**Fig. 1.**
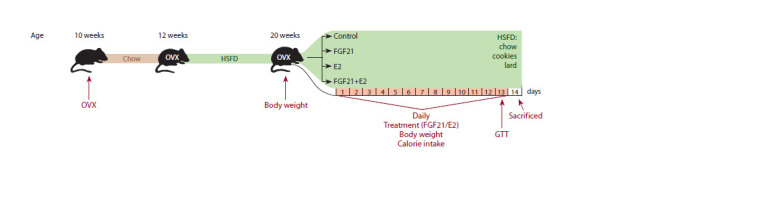
Scheme of the experiment. OVX – ovariectomy; GTT – glucose tolerance test; Chow – ad libitum access to commercial mouse chow; HSFD – high sweet fat diet (standard laboratory chow, sweet cookies, lard); Treatment – ovariectomized females with diet-induced obesity received fibroblast growth factor 21 (FGF21) at a dose of 1 mg per 1 kg of body weight subcutaneously and estradiol (E2) at a dose of 20 mg/animal orally separately or together for 13 days. Control females received subcutaneously phosphate buffered saline (PBS) and oil orally.

Female mice of C57BL/6J strain were kept at the vivarium for conventional animals of the Institute of Cytology and Genetics, Novosibirsk. The mice were housed at the 12-h/12-h light/dark regime (light from 07:30 to 19:30) at the ambient temperature 22–24 °C and free access to water and food. At the age of 10 weeks, all females were ovariectomized. To induce obesity, mice were fed with mixed diet, which consisted of standard laboratory chow, sweet cookies and lard, for two weeks after surgery. The animals consumed this diet for 8 weeks before and during the entire period (2 weeks) of drug administration (Fig. 1)

Mouse recombinant FGF21 (1 mg per 1 kg) dissolved in Phosphate Buffered Saline (PBS) or PBS itself were injected subcutaneously (V ≈ 200 μL) at the end of the light period (17:00–19:00) for 13 days. The expression and purification of mouse FGF21 were performed by Dr. Baranov, as described earlier (Makarova et al., 2021). Estradiol (E2 Sigma, USA, 20 μg/animal) dissolved in oil or oil itself were administered orally (V = 100 μL) at the same time as FGF21.

Obese ovariectomized female mice were randomly divided into four experimental groups (6–8 animals per group): (1) control females, which received vehicles (oil and PBS); (2) FGF21-females, which received FGF21 and oil; (3) E2- females, which received E2 and PBS; and (4) FGF21+E2- females, which received both FGF21 and E2.

The glucose tolerance test (GTT) was performed on day 13 of the experiment, after which each group received the last due treatment with drugs or solvents. One day after the last treatment, the females were weighed and decapitated (14:00– 16:00). E2 increased the uterus weight (43.5 ± 6.5 mg without estradiol (n = 15) vs 114.0 ± 8.6 mg (n = 12) after E2-treatment, p <0.001 Student test), indicating the effectiveness of the selected dose of the hormone. After decapitation, liver, subcutaneous (scWAT) white adipose tissue, abdominal (abWAT) white adipose tissue, and brown adipose tissue (BAT) were excised and immediately weighed. Blood and tissue samples were taken. Tissue samples for gene expression assays were immediately frozen in liquid nitrogen and stored until RNA isolation.

Diet. Standard chow was purchased from BioPro (Novosibirsk, Russia). The energy value of chow diet was 250 kcal/ 100 g. Pork lard and cookies were bought in a food store. The energy value of cookies was 458 kcal/100 g. The energy value of lard (subcutaneous fat) was 800 kcal/100 g. The number of calories consumed with each component of the diet was calculated as the weight of the component in grams multiplied by the energy value of the component. The percentage of calories consumed with each component of the diet (share of total) was calculated as the number of calories consumed by the female with the component divided by the total number of calories consumed and multiplied by 100.

Ovariectomy. The animals were anesthetized by an intraperitoneal injection of 2.5 % avertin (a mixture of 2,2,2-tribromethanol (Sigma-Aldrich Inc., USA) and 2-methyl-2-butanol (Sigma-Aldrich Inc.) in the volume 400 μL. Bilateral ovariectomy was performed through a skin incision in the lumbar region.

Glucose tolerance test (GTT). Before the test, food was removed from the animals at 08:00, and the test started at 15:00. Animals were injected with glucose (AO REACHEM, Moscow, Russia) intraperitoneally at the dose 1 g/kg body weight. Blood glucose concentrations were measured using a Lifescan One Touch Basic Plus glucometer (LifeScan Inc., Switzerland) before glucose administration (fasting glucose) and 15, 30, 60, and 120 minutes after glucose administration. The Area Under the Curve (AUC) was presented as mmol/L×hour.

Plasma assays. Concentrations of insulin, leptin, adiponectin, and corticosterone were measured using Rat/Mouse Insulin ELISA, Mouse Leptin ELISA (EMD Millipore, USA), Mouse Adiponectin/Acrp30 Quantikine ELISA (R&DSystems, USA), and CORTICOSTERONE rat/mouse ELISA (Xema Co. Ltd. in Moscow, Russia) kits, respectively. Concentrations of glucose, triglycerides, cholesterol, and free fatty acids were measured colorimetrically using Fluitest GLU, Fluitest TG, Fluitest CHOL (Analyticon Biotechnologies GmbH, Germany), and NEFA FS DiaSys (DiaSys Diagnostic Systems GmbH, Germany) kits, respectively. Fasting significantly increases endogenous FGF21 production and its level in the blood. As, the aim of this study was to compare the effect of prolonged FGF21 administration on metabolic parameters rather than the acute effects of FGF21, biochemical (and other) parameters were measured in fed animals.

Hepatic triglyceride content. Liver samples were homogenized in PBS (50 mg in 400 μL) and centrifuged at 1000 rpm. The supernatant was stored at –20 °C. Triglyceride levels were assayed using the Fluitest TG commercial kit (Analyticon Biotechnologies GmbH, Germany) according to manufacturer’s recommendations.

Relative quantitative real-time PCR. Total RNA was isolated from tissue samples using ExtractRNA kit (Evrogen, Moscow, Russia) according to the manufacturer’s recommendations. First-strand cDNA was synthesized using Moloney murine leukemia virus (MMLV) reverse transcriptase (Evrogen) and oligo(dT) as a primer. TaqMan gene expression assays (Applied Biosystems, USA) were used for relative quantitation real-time PCR. The genes tested involved fibroblast growth factor 21 (Fg f 21, Mm00840165_g1), peroxisome proliferator-activated receptor gamma coactivator (Ppargc1α, Mm01208835_m1), carnitine palmitoyltransferase 1A/1B (Cpt1α/β, Mm01231183_m1/Mm00487191_g1), acetyl- CoA carboxylase alpha/beta (Acacα/β, Mm01304257_m1/ Mm01204671_m1), insulin receptor (Insr, Mm01211875_m1), insulin receptor substrate 1/2 (Irs1/2, Mm01278327_m1/ Mm03038438_m1), protein-tyrosine phosphatase 1B (Ptpn1, Mm00448427_m1), pyruvate kinase (Pklr, Mm00443090_m1), glucokinase (Gck, Mm00439129_m1), glucose-6-phosphatase (G6pc, Mm00839363_m1), phosphoenolpyruvate carboxykinase (Pck, Mm01247058_m1), solute carrier family 2 mem-ber 2 (Slc2a2, Mm00446229_m1), solute carrier family 2 member 4 (Slc2a4, Mm00436615_m1), estrogen receptor 1 (Esr1, Mm00433149_m1), signal transducer and activator of transcription 3 (Stat3, Mm01219775_m1), peroxisome proliferator-activated receptor alpha/gamma (Pparα/γ, Mm00440939_m1/Mm00440940_m1), hormone-sensitive lipase (Lipe, Mm00495359_m1), adipose triglyceride lipase (Atgl, Mm00503040_m1), fatty acid synthase (Fasn, Mm00662319_m1), uncoupling protein 1 (Ucp1, Mm01244861_m1), deiodonase-2 (Dio2, Mm00515664_m1), corticotropin releasing hormone (Crh, Mm01293920_s1), agouti related neuropeptide (Agrp, Mm00475829_g1), neuropeptide Y (Npy, Mm01410146_m1), proopiomelanocortin (Pomc, Mm00435874_m1), leptin receptor (Lepr, Mm00440181_m1), klotho beta (Klb, Mm00473122_m1), cyclophilin A (Ppia, Mm02342430_g1), and beta-actin (Actb, Mm00607939_s1). Cyclophilin A and beta-actin were used as endogenous controls. The PCR and fluorescence detection were performed on an Applied Biosystems VIIA 7 Real-Time PCR System. Relative quantification was performed by the comparative threshold cycle (CT) method.

Statistical analysis. Each result is presented as the arithmetic mean ± SE. Two-way ANOVA with factors ‘FGF21’ (PBS or FGF21) and ‘E2’ (oil or E2) was used to analyze FGF21 and E2 effects on metabolic parameters with multiple comparisons by the post hoc Tukey test or Mann–Whitney U test in case of inequality of variances. Repeated measures ANOVA with factors ‘FGF21’, ‘E2’ and ‘Time’ (time after glucose load) was used to analyze the results of the GTT. Differences were considered significant at p < 0.05. Calculations were performed with the STATISTICA 10.0 software package (StatSoft Russia, Moscow, Russia).

## Results

Food intake, body weight, weights of liver and adipose tissues

**Fig. 2.**
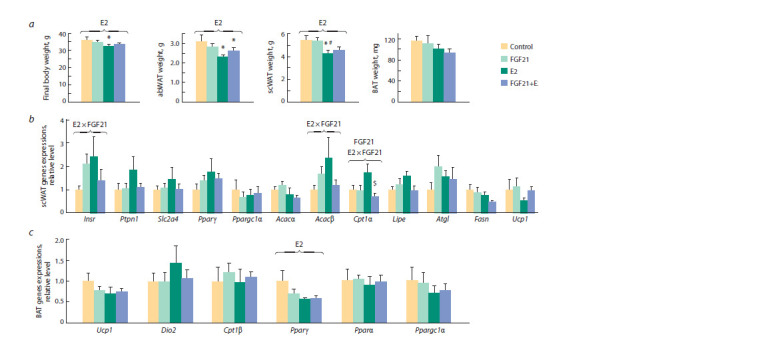
a, Body weight, the weight of abdominal (abWAT) and subcutaneous (scWAT) white adipose tissues and the weight of brown adipose tissue (BAT); b, c, the mRNA levels of genes regulating metabolism in scWAT and BAT, respectively. The ANOVA factors whose inf luence on the parameter is significant are shown above brackets (E2, FGF21, or E2 × FGF21). *vs Control, # vs FGF21, $ vs E2, post hoc Tukey test. Insr – insulin receptor; Ptpn1 – protein-tyrosine phosphatase 1B; Slc2a4 – solute carrier family 2 member 4; Pparγ – peroxisome proliferator-activated nuclear receptor gamma; Ppargc1α – peroxisome proliferator-activated receptor gamma coactivator; Acacα – acetyl-coenzyme A carboxylase alpha; Acacβ – acetyl-CoA carboxylase beta; Cpt1α – carnitine palmitoyltransferase 1A; Lipe – hormone-sensitive lipase; Atgl – adipose triglyceride lipase; Fasn – fatty acid synthase; Ucp1 – uncoupling protein 1; Dio2 – deiodonase-2; Cpt1β – carnitine palmitoyltransferase 1B; Pparα – peroxisome proliferator-activated receptor alpha coactivator.

**Fig. 3.**
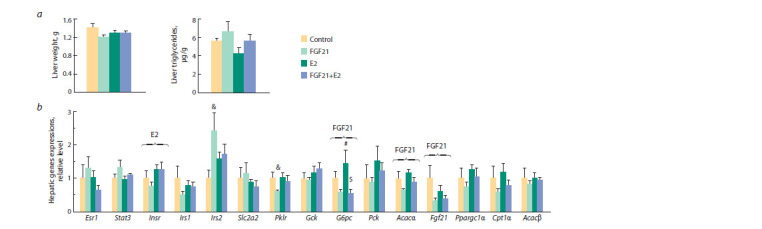
Liver: a, weight, triglyceride content; b, mRNA levels of genes regulating glucose and lipid metabolism. The ANOVA factors whose inf luence on the parameter is signif icant are shown above brackets (E2, FGF21, or E2 × FGF21). # vs FGF21, $ vs E2, post hoc Tukey test; &vs control, Mann–Whitney U test. Esr1 – estrogen receptor 1; Stat3 – signal transducer and activator of transcription 3; Insr – insulin receptor; Irs1/2 – insulin receptor substrate 1/2; Slc2a2 – solute carrier family 2 member 2; Pklr – pyruvate kinase; Gck – glucokinase; G6pc – glucose-6-phosphatase; Pck – phosphoenolpyruvate carboxykinase; Acacα – acetylcoenzyme A carboxylase alpha; Fgf21 – f ibroblast growth factor 21; Ppargc1α – peroxisome proliferator-activated receptor gamma coactivator; Cpt1α – carnitine palmitoyltransferase 1A; Acacβ – acetyl-CoA carboxylase beta

At the end of the experiment, E2-treated ovariectomized (OVX) obese females had lower weights of the body and of abdominal and subcutaneous adipose tissues (abWAT and scWAT, respectively) than oil-treated females ( p < 0.05, p < 0.05, and p < 0.01, respectively) (Fig. 2, a). Fibroblast growth factor 21 (FGF21) did not affect the parameters whether administered alone or with estradiol. There were no significant effects of the administration of E2, or FGF21, or both on the weight of brown adipose tissue (BAT), hepatic weight, or hepatic triglyceride content (Fig. 3, a, see Fig. 2, a).

**Fig. 4.**
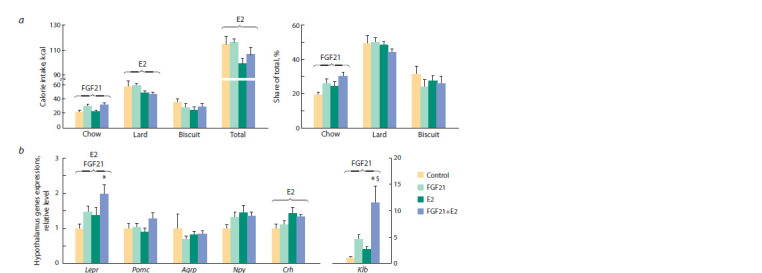
a, The number of calories consumed during the experiment (with each component and the total amount) and the percentage of each component (share of total); b, hypothalamic levels of mRNAs of genes associated with the control of food intake. ANOVA factors whose inf luence on the parameter is signif icant are shown above the brackets (E2, FGF21, or E2×FGF21).* vs Control; $ vs E2, post hoc Tukey test. Lepr – leptin receptor; Pomc – proopiomelanocortin; Agrp – agouti related peptide; Npy – neuropeptide Y; Crh – corticotropin releasing hormone; Klb – klotho beta.

Estradiol reduced the number of calories consumed with the high-fat component of the diet (lard) and the total number of calories consumed ( p < 0.01 and p < 0.05, respectively), but did not affect the contribution of various components of the diet to the calorie content of the food consumed (Fig 4, a). FGF21, regardless of E2, increased the number of calories consumed with standard chow and contribution of standard chow to the calorie content of the food consumed.

Thus, in obese OVX females, estradiol reduced body weight, apparently due to the decrease in WAT weight. Both drugs influenced the food preferences, and their effects were independent.

Insulin sensitivity, plasma hormone and metabolite levels

**Fig. 5.**
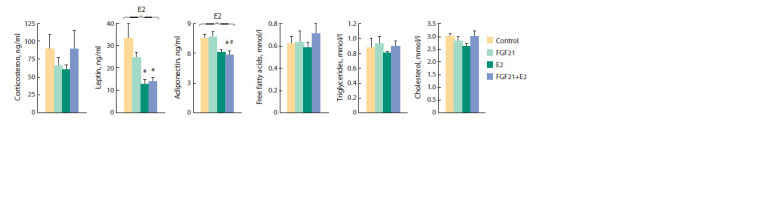
The level of hormones and metabolites in the blood plasma. ANOVA factors, whose inf luence on the parameter is signif icant, are shown above the brackets (E2, FGF21 or E2×FGF21).* vs Сontrol; # vs FGF21, post hoc Tukey test.

**Fig. 6.**
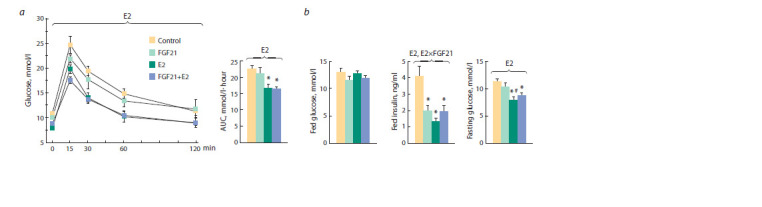
a, Glucose tolerance test: blood glucose levels before (0 min) and 15, 30, 60, and 120 minutes after glucose administration, and the area under the curve (AUC) of blood glucose levels; b, fed plasma levels of glucose and insulin, fasting blood glucose levels. ANOVA factors whose inf luence on the parameter is signif icant are shown above brackets (E2, FGF21 or E2×FGF21).* vs Сontrol, # vs FGF21, post hoc Tukey test.

In obese OVX females, there were no significant effects of separate or joint administration of drugs on the plasma levels of corticosterone, free fatty acids (FFA), triglycerides (TG), or cholesterol (Fig. 5). FGF21 had no effect on plasma levels of leptin or adiponectin, but estradiol reduced both ( p < 0.001). Estradiol reduced the plasma insulin and blood fasting glucose levels and increased glucose tolerance ( p < 0.001 in all cases) (Fig. 6, a, b). When administered separately, FGF21 also reduced the plasma insulin level ( p < 0.05, post hoc Tukey test). In females having received both drugs, insulin levels did not differ from those in FGF21- or E2-females, but they were significantly lower than in control females ( p < 0.05, post hoc Tukey test). Regardless of E2-treatment, glucose tolerance in females treated with FGF21 did not differ from control females and the fed plasma glucose level was lower than in control females, although the differences were below the level of significance ( p = 0.07).

Thus, estradiol increased insulin sensitivity in obese OVX females. FGF21, regardless of E2-treatment, did not affect glucose tolerance or the fasting glucose level, but lowered the fed glucose level. FGF21 also had a beneficial effect on the plasma insulin level, but this effect was recorded only in E2-untreated females

Metabolic gene expression

In scWAT, in obese OVX females, FGF21 and estradiol, when administered separately, increased the expression of insulin receptor gene (Insr) and acetyl-coA carboxylase beta gene (Acacβ, suppression of fatty acid oxidation) (see Fig. 2, b). In females who received both drugs, the expression of these genes was lower than in females who received one of them, and it did not differ from that in control females (influence of factor interaction p < 0.05 in both cases). In addition, when administered separately, FGF21 did not affect the expression of the Cpt1α gene for carnitine palmitoyltransferase 1α gene (activation of fatty acid oxidation) but suppressed its expression in E2-treated females (influence of factor interaction p < 0.05). In females receiving both drugs, the mRNA level of this gene was significantly lower than in E2-females ( p < 0.05, post hoc Tukey test) and did not differ from сontrol females.

There was no significant effect of separate or joint administration of drugs on gene expression in abWAT. In BAT, estradiol reduced the expression of the transcription factor peroxisome proliferator-activated nuclear receptor gamma (Pparγ) ( p < 0.05) (see Fig. 2, c). Regardless of the estradiol status, there were no effects of FGF21 on the expression of genes involved in thermogenesis and fatty acid beta-oxidation in BAT

Thus, in obese OVX females, the catabolic effect of estradiol was associated with its stimulating influence on the expression of Insr, Acacβ in scWAT and Pparγ in BAT. Despite the absence of the catabolic effect of FGF21, it exerted transcriptional effects in scWAT (stimulated the expression of Insr and Acacβ), but only in oil-treated females; in E2-treated females, the stimulating effect of FGF21 on gene expression was not manifested.

In the hypothalamus, changes in food consumption caused by the E2 action were associated with higher expression of the genes for leptin receptor (Lepr) and corticotropin-releasing hormone (Crh) ( p < 0.05 and p < 0.01, respectively) (see Fig. 4, b). The effect of FGF21 on food consumption was associated with an increase in Lepr expression ( p < 0.05), which was more pronounced in females receiving E2. In addition, FGF21 increased the expression of its own coreceptor, klotho beta (Klb) ( p < 0.01), and this effect was also more pronounced in females receiving E2.

Thus, the effect of drugs on the choice of food components was associated with a change in the hypothalamic expression of genes associated with the regulation of food consumption, and estradiol enhanced the transcriptional effects of FGF21

In the liver of obese OVX females, estradiol increased the expression of the insulin receptor gene (Insr) ( p < 0.05) (see Fig. 3, b). FGF21, regardless of E2-treatment, suppressed its own expression (Fg f 21) and the expression of genes asso- ciated with fatty acid synthesis and oxidation (acetyl-coenzyme A carboxylase alpha, Acacα, and carnitine palmitoyltransferase 1a, Cpt1α (tendency)), and with gluconeogenesis (glucose-6-phosphatase, G6pc) ( p < 0.05, p < 0.05, p = 0.08 and p < 0.01, respectively). When administered separately, FGF21 increased the expression of insulin receptor substrate type 2 gene (Irs2) and suppressed the expression of pyruvate kinase (Pklr, a key enzyme in glycolysis) ( p < 0.05, FGF21- females vs control females, Mann–Whitney U test in both cases). In females treated with both FGF21 and estradiol, the mRNA levels of Irs2 and Pklr did not differ from those in E2-females or control females.

Thus, independent transcriptional effects of FGF21 and estradiol in the liver were shown: FGF21-effects on G6pc, Acacα, and Fg f 21 expression and an E2-effect on Insr expression. However, the effects of FGF21 on Irs2 and Pklr expression manifested themselves only when the drug was administered without estradiol, and they were absent from animals receiving both drugs, which suggests an inhibition of the effects of FGF21 by E2.

## Discussion

In this work, we investigated whether the blood estradiol level modifies the pharmacological action of FGF21 in obese females. It is known that chronic FGF21 treatment improves many metabolic parameters in obese male mice; in particular, weight loss and lipoprotein profiles. It increases insulin sensitivity and energy expenditure, normalizes blood glucose and triglyceride levels, improves the liver state, and suppresses gluconeogenesis (Kharitonenkov et al., 2005; Coskun et al., 2008; Xu et al., 2009; Chau et al., 2010; Véniant et al., 2012; Fisher, Maratos-Flier, 2016; BonDurant, Potthoff, 2018). Also, FGF21 changes taste preferences, increasing protein consumption and reducing sugar consumption by male mice (Talukdar et al., 2016; Hill et al., 2019; Larson et al., 2019).

According to our data, estradiol given to OVX obese females reduced body and adipose tissues weights, the total number of calories consumed, fed insulin plasma level, and fasting glucose blood level and increased glucose tolerance. These observations agree with the generally accepted opinion as to the effect of estradiol on these parameters in female mice with obesity (Riant et al., 2009; Yan et al., 2019). We were first to demonstrate that the anorexigenic effect of estradiol in obese OVX females is due to the suppression of the consumption of a high-fat component of diet (lard), and this effect is associated with the activation of the expression of the corticotropin-releasing hormone gene (Crh) in the hypothalamus. However, some effects of FGF21 observed in obese males were not detected in obese OVX females. For example, FGF21, regardless of E2-treatment, did not affect body or adipose tissues weights; levels of lipids, leptin, or adiponectin in plasma; or the expression of genes associated with the regulation of thermogenesis in BAT, hypothalamic food consumption regulation, or with lipid metabolism in scWAT or in the liver. These results are consistent with data on the effect of FGF21 in non-ovariectomized obese females (Bazhan et al., 2019), and they suggest that there are sex-related factors other than estradiol that suppress the pharmacological effects of FGF21 in obese females.

We showed that the stimulating effect of FGF21 on the consumption of standard food in obese OVX females did not depend on E2-treatment, and it appeared to be similar to the FGF21 effect on food consumption in males. FGF21 is known to increase the consumption of protein (casein enriched with cystine) by males (Larson et al., 2019). In our experiment standard food had the maximum amount of protein, compared with lard and cookies. Both hormones, FGF21 and estradiol, affected the choice of food components, the influence of each was aimed to reduce the caloric content of consumed food; herewith, estradiol reduced the high-fat component of diet, and FGF21 increased intake of standard food. Both hormones increased the expression of leptin receptors (Lepr) in the hypothalamus. Genes for the hypothalamic neuropeptides proopiomelanocortin (Pomc), agouti related peptide (Agrp) and neuropeptide Y (Npy) are leptin targets (Cowley et al., 2001), and they are involved in the regulation of food consumption. We found no effect of FGF21 or estradiol on their expression, thus we assume that the activation of the hypothalamic expression of Lepr and, accordingly, enhane hypothalamic leptin sensitivity in response to the administration of FGF21 or estradiol do not mediate the effects of these hormones on food consumption. In this regard, the mechanism of FGF21 effect on food consumption remains unclear and requires further study, whereas the anorexigenic effect of estradiol is apparently due to the increase in hypothalamic Crh expression and activation of the CRF system. It should be noted that in the hypothalamus, FGF21 stimulated not only Lepr expression but also the expression of its own co-receptor klotho beta (Klb); the maximum expression of Lepr and Klb being recorded when drugs were administered jointly. The results indicate that the pharmacological use FGF21 can increase the sensitivity of the hypothalamus to regulatory factors, and estradiol can potentiate the central effects of FGF21.

We show that FGF21 administered to obese OVX females increases the expression of Insr and Acacβ in scWAT and Irs2 in the liver but suppresses the hepatic expression of glucose-6-phosphatase (G6pc), Pklr, Acacα, Cpt1α, and itself (Fg f 21). It is known that chronic administration of FGF21 to obese males increases the expression of Insr, Acacβ and suppresses the expression of Cpt1α in scWAT, and in the liver stimulates the expression of Insr and suppresses its own expression (Fg f 21) and the expression of Acacα and Cpt1α (Coskun et al., 2008; Fisher et al., 2011). Consequently, the transcriptional effects of FGF21 in obese OVX females not treated with estradiol were similar to those in obese males and beneficial. They were aimed at increasing insulin sensitivity in the liver and adipose tissue, and they contributed to a decrease in glucose and fatty acid plasma levels. In obese OVX females having received both drugs, the beneficial transcriptional effects of FGF21 persisted only in the liver: FGF21 suppressed the expression of G6pc, Acacα, Cpt1α, and itself.

When co-administered with estradiol, FGF21 suppressed the expression of Cpt1α in scWAT, but the effect of FGF21 on the expression of Insr and Acacβ in scWAT, Irs2 and Pklr in the liver was not pronounced. Consequently, the effect of FGF21 on hepatic G6pc expression, is independent of estradiol and is associated with a decrease in fed glucose plasma levels. We assume that these FGF21 effects (suppression of G6pc expression and lower plasma glucose level) were mediated by activation of hypothalamic Lepr expression, since the ability of leptin, affecting the activity of POMC neurons, to normalize blood glucose levels and increase insulin sensitivity in the liver has been shown (Berglund et al., 2012).

Thus, the study of the transcriptional effects of FGF21 in the liver, adipose tissue, and hypothalamus shows that there are different types of interaction between FGF21 and estradiol in regulating the expression of metabolic genes in obese OVX females: (1) FGF21 can act independently of E2, (2) estradiol may inhibit or enhance the effects of FGF21, and (3) the interplay of hormones can lead to mutual suppression of their effects, observed when they are administered separately. The ability of exogenous FGF21 to suppress the effects of estradiol in females suggests a possible adverse effect of pharmacological FGF21; in particular, on the female reproductive function.

What mechanisms may mediate the estradiol influence on the pharmacological effects of FGF21? FGF21 has been shown to bind to a receptor complex consisting of fibroblast growth factor receptor type 1 (FGFR1) and klotho beta co-receptor (Kurosu et al., 2007). In the hypothalamus, adipose tissue, and the pancreas, the receptor and co-receptor of FGF21 are expressed, as well as all types of estradiol receptors (Kurosu et al., 2007; Nadal et al., 2009; Fisher et al., 2011; Bian et al., 2019; Pan et al., 2019). These tissues are the target of FGF21 and estradiol. Estrogen receptor alpha and G-protein-coupled estrogen receptor are expressed in the liver (Palmisano et al., 2017), so the liver is the target of estradiol. The level of FGFR1 in the liver is very low (Fisher et al., 2011); however, some direct effects of FGF21 can be observed in the liver with its pharmacological administration, as a result of a large dose of the drug (Owen et al., 2015). The interaction of FGF21 and estradiol in the regulation of metabolic parameters may depend on the molecular mechanism regulating the expression of target genes, as well as on the type and level of receptors in the tissue, thus being tissue-specific. The molecular mechanism of this interaction requires additional study.

## Conclusion

To sum up, we state that ovariectomized obese females are resistant to the catabolic action of FGF21, and this resistance is not associated with the action of estradiol. The ability of FGF21 to increase the consumption of standard food and reduce blood glucose levels does not depend on estradiol either. However, FGF21 and estradiol appear to interact in the regulation of gene expression and blood insulin levels: (i) estradiol can suppress the transcriptional effects of FGF21 in the liver and potentiate its effect in the hypothalamus; (ii) in adipose tissue, the interaction of FGF21 and estradiol can suppress the activating effect of each of the drugs observed with separate administration or contribute to the manifestation of the inhibitory effect of FGF21; and (iii) in E2-treated animals, FGF21 exerts no inhibitory effect on the blood insulin level.

Estradiol-dependent effects of FGF21 can manifest themselves differently in male and female bodies, different in estrogen activity. Thereby, they determine the sexual dimorphism of the pharmacological effects of FGF21 in obese animals.

## Conflict of interest

The authors declare no conflict of interest.
